# The EPIRARE proposal of a set of indicators and common data elements for the European platform for rare disease registration

**DOI:** 10.1186/2049-3258-72-35

**Published:** 2014-10-13

**Authors:** Domenica Taruscio, Emanuela Mollo, Sabina Gainotti, Manuel Posada de la Paz, Fabrizio Bianchi, Luciano Vittozzi

**Affiliations:** National Centre for Rare Diseases, National Institute of Health, Rome, Italy; Institute of Rare Diseases Research (Instituto de Investigación de Enfermedades Raras - IIER), Instituto de Salud Carlos III (Instituto de Salud Carlos III - ISCIII), Madrid, Spain; RDR and Consortium for Biomedical Research in Rare Diseases (Centro de Investigación Biomédica en Red de Enfermedades Raras -CIBERER), Madrid, Spain; Institute of Clinical Physiology, National Council of Research, Pisa, Italy

**Keywords:** Registries, Common data elements, European platform, Rare diseases, Patient registration, EPIRARE

## Abstract

**Background:**

The European Union acknowledges the relevance of registries as key instruments for developing rare disease (RD) clinical research, improving patient care and health service (HS) planning and funded the EPIRARE project to improve standardization and data comparability among patient registries and to support new registries and data collections.

**Methods:**

A reference list of patient registry-based indicators has been prepared building on the work of previous EU projects and on the platform stakeholders’ information needs resulting from the EPIRARE surveys and consultations. The variables necessary to compute these indicators have been analysed for their scope and use and then organized in data domains.

**Results:**

The reference indicators span from disease surveillance, to socio-economic burden, HS monitoring, research and product development, policy equity and effectiveness. The variables necessary to compute these reference indicators have been selected and, with the exception of more sophisticated indicators for research and clinical care quality, they can be collected as data elements common (CDE) to all rare diseases. They have been organized in data domains characterized by their contents and main goal and a limited set of mandatory data elements has been defined, which allows case notification independently of the physician or the health service.

**Conclusions:**

The definition of a set of CDE for the European platform for RD patient registration is the first step in the promotion of the use of common tools for the collection of comparable data. The proposed organization of the CDE contributes to the completeness of case ascertainment, with the possible involvement of patients and patient associations in the registration process.

**Electronic supplementary material:**

The online version of this article (doi:10.1186/2049-3258-72-35) contains supplementary material, which is available to authorized users.

## Background

The European Union (EU) acknowledges the relevance of registries as key instruments for developing rare disease (RD) clinical research, improving patient care and health service (HS) planning [1, 2]. The European Commission has funded the EPIRARE and other projects on EU patient registration, and stated that its strategic objective is the creation of the European Platform for RD patient registration (RDR), providing common services and tools for the existing (and future) rare disease registries in the EU [3]. The EPIRARE project (“Building Consensus and Synergies for the EU Registration of Rare Disease Patients”, http://www.epirare.eu), studied a model for this platform [4] and concluded that it should have an important role in improving standardization and data comparability and, where useful, supporting the set up of new registries. Actual data collection should be limited to diseases for which disease-specific registries are not sustainable or for which there is no specific research interest. This article presents the results of the EPIRARE project defining a set of common data elements (CDE) for the European RDR Platform. Although European or wider data sharing would be desirable to increase the power of data analyses, the reference to the European RDR Platform CDE by new and existing registries will impact positively on data and indicator comparability independently of data sharing, which might be dramatically hampered by the next regulation on personal data protection, which is currently under discussion in the EU Parliament.

## Methods

In line with recommended methodologies [5], at first a reference list of registry-based indicators was defined, starting from the indicators identified by the EUROPLAN project [6] and the EU Rare Disease Task Force (RDTF) [7]; some indicators were slightly modified or added, in consideration of the opinions expressed by the RDTF experts and of the information needs of the identified stakeholders as resulting from the surveys [8, 9] and consultations [4] carried out during the EPIRARE activities. The experts who reviewed the cited RDTF document and the EUROPLAN Working Group on indicators are reported in the cited documents. The process of selection of the addressees of the EPIRARE surveys and consultations is reported in the cited references. More detailed indications of the respondents and the EPIRARE advisory board members are presented, respectively, in the deliverables and partners sections of the EPIRARE project website (http://www.epirare.eu). The resulting set of variables necessary for the computation of these indicators was compared with the information regarding institutional initiatives for national RD registries already established or in preparation which were notified to EPIRARE from experts in Belgium, Bulgaria, France, Germany, Italy and Spain in order to have the highest consistency among EU registries. The definitions and formats of the selected variables were kept as far as possible similar to the data elements used in the US NIH Global Rare Disease Registry to facilitate any possible collaborative work. Finally, the peculiarities of some variables and of their collection were also considered to elaborate the proposed organization of the CDE set.

## Results and discussion

### The set of reference indicators

The set of rare disease indicators, which were used in this study as reference for the selection of the CDE, is reported in “Additional file 1”. These indicators span from disease surveillance, to socio-economic burden, HS monitoring, research and product development, policy equity and effectiveness. The indicators mentioned in the research area have generic definitions, but represent many possible indicators which may be defined for specific goals, mostly depending on clinical data. “Additional file 1” reports also the variables which were considered necessary for the computation of each indicator.

### Specific features of groups of variables

Besides the computation of sound platform indicators and other information outputs, some variables have a particular importance for the best use of registry data. These comprise a) an unambiguous universal patient coding; b) the variables allowing indicator analysis by diagnosis, geographic location of the patient and health care services used by the patient; and c) variables allowing the ethical processing of patient data, including his/her willingness to participate in research.

### The set of common data elements and its organization

Following the results of the analysis described above and in line with the cluster analysis of the scope of data collection by registries with different aims (Santoro M, Coi A, Lipucci Di Paola M, Gainotti S, Mollo E, Taruscio D, Vittozzi L, Bianchi F: A classification of the Rare Disease Patient Registries aimed at identifying different informative needs, submitted), the data elements were organized in three different domains (Table 1). The first domain aims mainly at facilitating the completeness of case notification and includes the case identification, the geographical location of the patient and of the services involved in the patient treatment, as well as information on the patient position regarding his/her participation in research. This is the minimum information necessary to characterize the case and most of it is collected in usual medical practice; therefore, it is proposed as the mandatory set of data elements. It is made of data which are in the knowledge of the patient (or their family) and which can be entered without the involvement of physicians or the health services which follow the patient. Although validation of patient-reported data may be recommended before its inclusion in the database, this additional source, by promoting the case notification to registry holders, may increase the sensitivity of the registration system and allow also sensitivity estimates. Finally, this data set provides information on the patient distribution and problem dimension, and is of use for HS and clinical trial planning, for the prioritization of product development and for patient advocacy. The variables necessary to compute a univocal patient code (EU GUID) have been selected following the results of Johnson et al. [10]. However, to improve coding accuracy in a global context with multiple languages and alphabets, it is considered necessary that EU registry sources collect two additional elements for the EU GUID elaboration: the country of birth, which is already collected in the US-GRDR [11], and the national unique identification code.

**Table 1:** **The EPIRARE set of common data elements for the European RDR platform**

	COMMON DATA ELEMENTS collected in the EPIRARE platform (elements in bold require longitudinal data collection)	ANNOTATIONS regarding the data elements; ***Where indicated***: DEFINITIONS and FORMATS	REASON
*Domain 1) Case characterization essentials*			
Case notification - Mandatory data	EU Global Unique Identifier (EU GUID)	This code is elaborated from the following data elements:	Unambiguous patient coding (to be processed according to legal provisions) is necessary to keep the integrity of the database and avoid duplication of records.
		• Patient given name: DEFINITION: “First name of patient as recorded in birth certificate, passport or identity card”; FORMAT: full name, not initials	
		• Patient family name (at birth): DEFINITION: “Family name of patient as recorded in birth certificate, passport or identity card”; FORMAT: full name, not initials	The National Unique Identification Code increases the accuracy of the EU GUID in case of names in foreign languages. It could be an optional part of the encrypted code.
		• Patient sex: see definition below	
		• Patient date of birth: see definition below	
		• Patient city of birth: see definition below	
		National Unique Identification Code	
	Patient sex	DEFINITION: “Patient’s physical sex at birth”; PERMISSIBLE VALUES: male, female, other (in any format)	Allows studies of sex-related differences in the disease epidemiology and clinical features
	Patient date of birth	DEFINITION: “Date of patient’s birth recorded in birth certificate, passport or identity card”; FORMAT: complete date (year, month, day) in any format	Allows studies of age-related disease features.
		For privacy reasons, depending on the time course of the disease, this data is to be communicated to the platform at the appropriate level of precision (only month and year or complete)	
	Patient city of birth	DEFINITION: “Name of city/town/village where the patient was born as it appears on the birth certificate, passport or identity card”; FORMAT: full name of city.	This data may be communicated to the platform only for some specific diseases for studies of health determinants.
		For privacy reasons, this data is to be communicated to the platform with the appropriate level of precision (e.g. mapped to the province, or to postal code). Moreover, it is important that geographical names are mapped to the INSPIRE identifiers [12]. This will enable the link with platforms organized around environmental spatial information, such as environmental pollution databases. This may offer an additional opportunity to indicate the place with an appropriate granularity to comply with privacy needs.	
	Patient country of birth	DEFINITION: “Name of country where the patient was born as it appears on the birth certificate, passport or identity card”; FORMAT: full name of country	Increases the discriminatory power of the EU GUID in global registries
	Diagnosis	Multiple coding according to current relevant classification systems is recommended while waiting for a general reference classification of rare diseases	Attribution of a disease to the case
	**Patient city of residence**	DEFINITION: “Name of city/town where the patient usually lives”; FORMAT: full name of city	Attribution of the case to a geographic area; prevalence, incidence, mobility
		For privacy reasons, this data is to be communicated to the platform with the appropriate level of precision (e.g. mapped to the province, or to postal code). Moreover, it is important that geographical names are mapped to the INSPIRE identifiers [12]. This will enable the link with platforms organized around environmental spatial information, such as environmental pollution databases. This may offer an additional opportunity to indicate the place with an appropriate granularity to comply with privacy needs.	
	**Patient country of residence**	DEFINITION: “Name of country where the patient usually lives”; FORMAT: full name of country	Attribution of the case to a geographic area; prevalence, incidence, mobility
	**ID Treatment Centre**	Treating Centre Full name/code; contact data are optional to improve identification	Attribution of the case to the treating setting
	**Treating Centre City-Town**	FORMAT: full name of city	Attribution of the centre to a geographic area; patient mobility for treatment; planning research/clinical trials
		It is important that geographical names are mapped to the INSPIRE identifiers [12].	
	**Current and past participation in clinical trials**	Yes/No	Planning research/clinical trials
	**Patient willingness to be contacted to participate in a future clinical trial**	Yes/No	Planning research/clinical trials
	**Patient willingness to be contacted about donating biological samples**	Yes/No	Planning research/clinical trials
	**Patient consent**	based on graduated consent forms	
	**Patient contact**	contact details; preferred means of contact (including via intermediary physician); language	
*Domain 2) Determinants and services*			
Case characterization	Other cases in the family	Yes/No (If Yes: degree of kinship)	Socio-economic burden of disease
	Healthy carriers in the family	Yes/No (If Yes: degree of kinship)	
	Case parents are consanguineous	Yes/no	Contribution of consanguinity
	Genetic features of the patient	Gene-HGNC Gene Symbol	Link to genetic research platforms; patient cohort selection
		Chromosome number	
		Nucleotide sequence analyzed and reference sequence systems with accession and version number	
		Variant description in HGVS format	
		Variant description in other formats	
History of diagnosis	Date of first symptoms onset	DEFINITION: “Date when patient first began experiencing symptoms or signs of the rare disease”; FORMAT: complete date (year, month, day) in any format	Age at onset; time to diagnosis
	Date of first contact of patient with the public Health Service	Date of the first time the patient requested a medical visit of the health service with reference to the symptoms of the diagnosed rare disease	Time to diagnosis
	ID Centre/physician referring the patient to the RD centre	Centre/Physician Full name/code; contact data are optional to improve identification	Integration of RD centres in the general Health Service
	Date of current diagnosis	DEFINITION: “Date when the current rare disease diagnosis was made” FORMAT: complete date (year, month, day) in any format	Time to diagnosis; life expectancy at diagnosis
	Status of current diagnosis	Suspected-confirmed	Diagnostic patterns; time to diagnosis; life expectancy at diagnosis
	Methods used for current diagnosis	List to be defined	Diagnostic patterns
	ID Centre which made diagnosis	Centre Full name/code; contact data are optional to improve identification	
	Centre which made diagnosis City-Town	FORMAT: full name of city	Patient migration for diagnosis
		It is important that geographical names are mapped to the INSPIRE identifiers [12].	
	Patient referred after positive neonatal screening result	Yes/no	Sensitivity of neonatal screening tests; effectiveness of neonatal screening program
Treatments and services	**Current orphan drug treatment**	DEFINITION: “A list of all current orphan drugs that a patient is currently taking”; FORMAT: name of all active ingredients (ORPHANET list)	
	**Current off-label drug treatment**	DEFINITION: “A list of all current drugs (different from orphan drugs) that a patient is currently taking”; FORMAT: name of active ingredients	
	**Current drug treatment**	DEFINITION: “A list of all current drugs (different from orphan drugs) that a patient is currently taking”; FORMAT: name of active ingredients	
	**Hospitalizations**	DEFINITION: “Cumulative number of patient’s admissions to the hospital due to the rare disease”; FORMAT: number	
	**Transplantations**	Yes/No (If yes: date of transplantation; tranplant material)	
	**Surgeries**	Yes/No (If yes: date of surgery; ID code of Surgery)	
	**Current dietary regimens prescribed as treatment**	Yes/No (If yes: type of regimen)	
	**Current assistive devices**	Yes/No (If Yes: Type of assistive devices used by patient; ID Code of type of device.	
	**Other treatments**	If Yes: Type/Code of treatment; indicate if current or date of administration	
	**Biomaterial donated**	(Yes/no); If Yes: list to be defined (e.g. Tissue or body fluid or other specifications)	Planning research/clinical trials
	**ID Biobank where the biological sample is stored up**	Biobank Full name/code; contact data are optional to improve identification	Link to Biobanks; planning research/clinical trials
	**(if the biobank storing the sample is not known) ID Centre which sampled the biomaterial**	Sampling Centre Full name/code; contact data are optional to improve identification of the centre	Link to Biobanks; planning research/clinical trials
*Domain 3) Outcomes*			
	**Patient vital status** (and date of death)	Live/Dead (If Dead: complete date of death (year, month, day) in any format Required Sources: National Death Registry or National Population Registry	
	**Education level**	Values from 0 to 8, based on the ISCED 2011 classification	Studies of socio-economic burden. Comparison and matching of patient populations from different data sources on the basis of socio-economic data. Applicable to individuals from early childhood.
	**Occupational status**	Self-defined current economic status (PL031 EU-SILC Target Variable): 11 possible values. (http://epp.eurostat.ec.europa.eu/portal/page/portal/income_social_inclusion_living_conditions/documents/tab/Tab/Personal%20data%20-%20labour.pdf)	Studies of socio-economic burden. Comparison and matching of patient populations from different data sources on the basis of socio-economic data.
			Applicable to individuals more than 16 year old.
	**Patient HRQoL index score**	Patient health-related quality of life (HRQoL) generic questionnaires with calculation of QALYs or the utility score	assessment of the Health-related Quality of Life; QALYs; equitable decision-making
	**Comorbidity**	DEFINITION: “Other diseases observed in the patient”; FORMAT: ICD10 (multiple coding in case that other RD are observed)	
	**Remarkable or unusual symptoms**	Remarkable or unusual symptoms, including adverse effects of treatments, and their severity (based on a 5-degree scale).	

The second domain of the platform data elements aims at characterizing the patient risk factors and at monitoring and planning the operation of the health services. It extends the patient characterization with genetic data and with data regarding his/her health status and familial information. Moreover, this domain includes data regarding the history and status of diagnosis and treatments. This information can be collected from a variety of sources and requires specific methodological expertise for the data collection and use for HS research.

The third domain aims at supporting outcome analysis. It includes data of patient death; of health-related quality of life (HRQoL), education level attained and occupational status for an integrated assessment of the patient condition;, and of co-morbidity and other symptoms, which are observed and may be associated with the case disease and treatments. The assessments of the education level attained, occupational status and HRQoL, which are not in the usual interest of pathology registries, require the administration of a short questionnaire. These data are extremely important since many RD are not impacting on the lifetime and can serve many purposes, from patient-centered description of the disease course, to monitoring the impact of policies and best practices, to provide a basis for patient advocacy actions and to equity decisions based on the burden of disease and on assessments cutting across all diseases. The variety of disease specific clinical data and of their observation conditions prevents, at present, its collection within a set of CDE, although they are central in the interest of clinicians and in the scope of many registries. The EPIRARE project suggested that the European RDR Platform could host a section of metadata of the clinical observations collected by individual registries, in order to facilitate traceability of existing data and contacts with registries collecting relevant data.

## Conclusions

The definition of a set of CDE for the European RDR Platform has different bearings for the databases of registries in comparison to the database in the European RDR Platform. For registries, this set of CDE is not to be considered as the fixed structure of a common database to be used by all registries regardless of their purposes. Rather, it intends to provide “building blocks” for the construction of registries for a variety of purposes. Therefore a registry should select, beside the mandatory set (domain 1 data), the data elements, which are necessary to compute the indicators relevant for the purposes it intends to pursue, and collect the corresponding data according to the definitions and formats proposed. Moreover, in case that the registry intends to collect outcome data, it is recommended that all the data indicated in domain 3 are collected. Finally, it is up to the registry the choice to collect additional data, not included in the set of CDE, for more detailed or specialized observations which are necessary for its own specific study purpose, such as treatment-specific features or disease-specific clinical data. Therefore the adoption of the European RDR Platform CDE has the main aim to promote the collection, according to common specifications, of data necessary to compute indicators which are both relevant to the purpose of the registry and key for more general purposes regarding RD, the achievement of which may require indicator and data comparability. The actual practice of collection of this data according to the specifications proposed by EPIRARE, the feasibility of adaptation to the proposed specification and the further usability of data already collected has been studied and is the subject of a manuscript in preparation. Moreover, this practice will contribute, in case that this will be allowed by the next regulation on data protection, to the interoperability and data merging among different registries. Within a scenario of feasible data sharing, the European RDR Platform could accommodate and use the relevant data communicated by registries for the computation, as far as feasible, of indicator values from a wider evidence base, or to support the collection of data tailored to the specific features of many different diseases. For these aims, its database should necessarily consist of the full set of CDE and, likely, of additional metadata to facilitate traceability of existing data and contacts with the sources of data, including more detailed or specific observations. The definition of a set of CDE for the European RDR Platform is the first step in the promotion of the use of common tools for the collection of comparable data of RD patients. The next step in this process is the definition of common references for those data which can be entered following different coding systems, catalogues or measuring scales. The standards and terminologies to be used in the platform should be agreed with clinical and epidemiological experts and, possibly, involving representatives of EU national information systems.

## Electronic supplementary material

Additional file 1: Selected platform indicators and measures, with the combinations of variables considered necessary to calculate them.(PDF 241 KB)
