# Supplementary material for: The EPIRARE proposal of a set of indicators and common data elements for the European platform for rare disease registration
Source: Arch Public Health. 2014 Oct 13;72:35. doi: 10.1186/2049-3258-72-35 (PMC4210567; doi:10.1186/2049-3258-72-35)
Supplement: Supplementary file 1 — Additional file 1: Selected platform indicators and measures, with the combinations of variables considered necessary to calculate them.(PDF 241 KB) [file 13690_2014_5051_MOESM1_ESM.pdf]

Additional file 1 – Selected platform indicators and measures, with the combinations of variables considered necessary to calculate them

	Registry-based indicators and other measures (further data selections by disease, time period and geographic area are implicit)	Combination of variables considered necessary for the computation of the indicator (diagnosis is always considered) (in bold: patient follow-up is required)	Remarks
a) Contribution of RD to morbidity/mortality			
	Prevalence, per disease and global*	date of diagnosis; **patient city of residence**	
	Incidence, per disease and global*	date of diagnosis; date of death; patient city of residence	
	Age at death**	Date of birth; Date of death;	Required Source: National Death records;
	age at disease onset**	Date of birth; date of onset	
	life expectancy at diagnosis***	date of diagnosis; Date of death;	
	disability profile**	**Disability scores** (health profile from a disability generic questionnaire)	Required: agreed reference generic (modular) disability questionnaire among those available; additional use of a reference validated questionnaire specific for the disease or group of diseases is welcome, if available, but it does not support the set of Common Data Elements.
	Hospital admissions*	**Hospitalization**	
	number and types of transplantations**	**transplantation data**	
	number and type of surgeries recorded***	**Surgeries data**	
	number of RD actually diagnosed (and recorded) per Country and per Centre§	diagnosis	
b) Socio-economic impact			
	Health-related Quality of Life index**	**HRQoL index (utility) score**	Required: agreed validated reference generic QoL questionnaire among those available.<br>Required source: patients, possibly assisted by their association or by physician/nurse
	number of patients treated per OD per year**	**OD treatment**	
	other cases in the family**	Diagnosis of the relative case	
	healthy carriers in the family**	Genetic features of the family	
	Contribution of consanguinity	case parents are consanguineous	

	Impact on education***	highest education level attained	
	Impact on occupation***	occupational status	
c) (Availability of appropriate) Health Services			
	effectiveness of neonatal screening programs§ (sensitivity)	neonatal screening results	
	effectiveness of neonatal screening programs§ (positive predictive value)	neonatal screening results	Required Source: registries including suspect patients
	time from disease onset (where reliably feasible) to confirmed diagnosis**	Date of onset; date of diagnosis	
	time from 1st report to the health service to confirmed diagnosis**	Date of first report to the health service; date of diagnosis	
	ODs actually used (and recorded)§	**OD treatment**	
	number of patients treated per ODs§	**OD treatment**	
	patients' mobility for diagnosis***	Diagnosis centre city; patient residence city	
	patients' mobility for treatment***	**Treating centre city; patient residence city**	
	Number and directory of centres actually making diagnosis§	ID of Diagnosis Centre	
	Activity of centres actually making diagnosis (diseases diagnosed and number of diagnoses per year)§	date of diagnosis; ID of Diagnosis Centre	
	Number and directory of treatment centres§	**ID of Treatment Centre**	
	Activity of treatment centres (diseases treated and number of treated patients per year)§	**ID of Treatment Centre**	indicated by the RDTF WG as a possibly better indicator; requires the date of record update
d) Information, research, technology development			
	indicators supporting cohort selection and patient recruitment for CT***	One example of many possible: genetic variation; **patient's willingness to participate; patient contact information; comorbidity.** Additional/alternative **disease-specific clinical data** may be necessary.	
	indicators based on disease-specific clinical data (e.g. clinical care benchmarks)	E.g.: Combinations of **disease-specific clinical data** and **treatment data**	
e) Equity, EU initiatives			
	no specific indicators, but comparisons of the defined indicators calculated in the relevant subgroups of patients		additional data elements required: patient residence Country; diagnosis centre ID (and city and Country); treatment centre ID (and city and Country);

	Health-related Quality of Life index**	**HRQoL index (utility) score**	Required: agreed validated reference generic QoL questionnaire among those available.<br>Required source: patients, possibly assisted by their association or by physician/nurse
*These indicators were considered by the RDTF particularly important for surveillance of status and trends			
§ EUROPLAN indicators			
**These measures are the proposed alternates to the indicators considered by the RDTF			
***These are additional measures considered by EPIRARE			
